# Understanding the Impact of Integrated Care

**DOI:** 10.5334/ijic.11901

**Published:** 2026-09-21

**Authors:** Nicholas Goodwin

**Affiliations:** 1 Health Policy, Centre for Research in Health Systems Performance, Yong Loo Lin School of Medicine, National University of Singapore, Singapore

**Keywords:** evaluation, evidence, impact, integrated care, theory of change, value

It has often been observed that the field of integrated care lacks knowledge and evidence of impact, both on how it effects people’s experiences and care outcomes, but also on its contribution to health and care system performance and sustainability [1]. A recent review of articles published in IJIC revealed that measuring the impact of integrated care is challenging and that our ability to demonstrate solid and unbiased evidence that integrated care works, and in what context, is weaker than it should be [2].

A part of the problem is the recognition that, in most cases, the application of integrated care represents a complex innovation that requires development and adaptation within the context of its application. As a result, no two integrated programs are exactly alike, meaning that there is no ‘all inclusive’ framework or set of methods that can adequately capture the impact of integrated care _ both within specific programs, but also to compare between them [3].

More fundamentally, measuring the impact of integrated care requires a multifaceted approach, considering various perspectives across different contexts and using diverse measures [4]. Established thinking might focus on the collective purpose of integrated care as means to improve care experiences, care outcomes, and cost-effectiveness of care (the basic ‘value’ proposition). However, impact may also encompass many other elements such as workforce capability and well-being, co-production partnerships with people and communities, ability to address health equity and the social determinants of ill-health, quality of life, and the many aspects of system sustainability, including environmental sustainability.

How, then, should we judge impact and from who’s perspective?

Perhaps the most fundamental point is that the measures one chooses to assess an integrated care program need to be driven by both the logic of the program itself, and the values of those both delivering and benefiting from it. For example, the recent paper by Tinella et al (2026) that focused on integrated care for the homeless argued that it was crucial to understand people’s preferences to identify what matters most to them to both align services, but also to evaluate them [5]. They used participatory methods (discrete choice) to achieve this. Such findings reinforce other recent work describing the importance of embedding co-creation principles in bringing together the design and implementation elements at one with evaluation practices to both enable continuous learning and support the ongoing growth and sustainability of integrated care programs [6].

The broader implication from the above is that a ‘theory of change’ approach to integrated care is needed. By bringing stakeholders together in a participatory process to understand how and why change is likely to occur, shared goals and desired outcomes can be created. This provides a logical framework for measuring impact and, in turn, should enable monitoring and evaluation processes to promote shared learning.

The scoping review by Saari et al (2026) gives an indication of the likely limited range of evidence that may emerge when such approaches are *not* addressed [7]. In using the Quintuple Aim to examine the value of integrated home and community services, and of the 47 peer-reviewed studies included in their review, they found that zero focused on health equity and just two on population health outcomes. Conversely, impact assessments focused almost entirely on costs and utilisation of hospital (n = 36) and emergency services (n = 22). The authors demonstrate that most of the programs were founded on value-based propositions, but that the emphasis on cost-related outcomes reflected prevailing austerity and value-based rhetoric shaping research funding and system priorities. They conclude that future research should embrace a more diverse value-based approach linked to a learning health system model.

There have been many advances in how to strengthen and measure research impact to support research translation in complex real-world settings. A good example from Australia is the Framework to Assess the Impact from Translational Research (FAIT) that acts as a multi-dimensional evaluation methodology using program logic to map out the entire research pathway from initial project aims to final end-user adoption, tracking impact metrics over time [8]. In essence, it encourages the use of a basket of indicators to derive a wider understanding of the value of an implementation program from a clear understanding of how things were planned, designed and implemented in the context of their application.

This thinking is not new to integrated care. A decade ago, considerable effort internationally was put into the development of indicators that could be used to judge the impact of integrated care [9]. Such work emphasised how a theory of change approach should be embraced that identified the core aims of integrated care in terms of who and what the interventions involved are seeking to influence and the range of desired outcomes that should result. This work also stressed the importance of participatory co-design and value creation to build engagement and ownership of the process to drive continuous quality improvement.

Increasingly, this thinking has translated into how economic evaluations in integrated care may be undertaken. For example, the recent review paper by Hobden et al (2026) examined methods to conduct economic evaluations of cross-organisational care pathways [10]. It concluded that the complexities of working across care organisations require evaluators to carefully consider the context, design, implementation and expected impacts of integrated care. Hence, if evaluators want to gain an overall picture of the value of integrated care – one that aligns with a program’s goal and those engaged with it – approaches such as multi-criteria decision analysis or cost consequence analysis maybe most suitable. Echoing the deliberations from the work of Tsiachristas et al [11], their key learning is that impact cannot be simplified into one single metric, but that a basket of relevant indicators is required to support multi-agency deliberations. Hence, the appropriateness of methods to support economic evaluations are dependent on the purpose of the program and the priorities of its key stakeholders. This represents a ‘value for investment approach’ in which economic thinking should be embedded into the design or modelling phase of complex service innovations [12].

By embedding research throughout the integrated care journey, it may also be more possible to effectively deploy realistic evaluation methods. Realistic evaluation has long been seen as a most promising way to understand how outcomes and impact emerge in integrated care initiatives [13,14], though has often been criticised for being descriptive and retrospective. A recent review of realistic evaluation pointed to the need for greater rigour in how such evaluations are formulated, specifically to ensure that it fits within the theory of change of the program it is seeking to assess and uses metrics and measures that are appropriate to test the hypotheses from that theory [15]. In other words, grounding evaluations of integrated care in realistic principles can only enhance causal influence and facilitate learning on impact if they are directly embedded in the program logic from the outset, rather (as is more usual) as a guiding framework to retrospectively interpret results.

The emerging answer, then, as to how we can best judge impact and from who’s perspective appears to be predicated on integrated care programs working through a co-produced theory of change that identifies the logic behind their initiatives and how these meet the priorities and values of the stakeholders involved. By creating a shared set of measurable outcomes that might be achieved over time, appropriate methods may then be constructed to provide both an understanding of impact, insights into how and why these resulted, and guidance to support future activities.

The obvious problem to such thinking is that we know that most integrated care programs are neither based on co-productive processes nor focused on co-creating a program logic to underpin an understanding of how things may be achieved. Of those that do, the program logic can often be underdeveloped or flawed and end up placing unreasonable expectations on a service. Too often, understanding outcome is not embedded from the outset but retrospectively fitted in ways that may ignore the values and objectives that were part of the original design. Evaluations, too, usually arrive late to the party, so contributing to a retrospective rather than prospective approach. Even where embedding evaluation from the start of the process is recognised, the requirements for such investment are often prohibitive (both in terms of resources and skills) leading to cut-down versions that prioritise funder-driven priorities and timescales.

These evaluation challenges go well beyond the field of integrated care but speak to the need to build a translational research agenda where ongoing evaluation is seen as a necessary component of any existing innovation to promote learning and support continuous improvement. As Alvarez and Aldasoro (2026) put it, ‘*any evaluation of … integrated care services and programs require a high degree of dexterity, resourcefulness, and inventiveness to navigate through an inherently complex learning process. It means crafting a research-oriented exercise, a policy-oriented exercise, and a change management exercise—all at once*’ [16, p928]. It is perhaps not surprising, then, that the evidence for the impact of integrated care is seemingly deficient given the lack of thought or sophistication in how evaluation could be embedded in the process. Integrated care presents deeply rooted challenges in evaluating something that is dynamic and relational, and as a result we should not be surprised that evidence presents limited, uneven and variable results.

It is tempting to conclude that longer, recursive and better designed evaluations that are fully embedded in the implementation process of integrated care programs and which answer questions directly related to their purpose is required. Yet, the dawning reality is that our current evaluation needs demand more time and resources than health and social care systems can reasonably be expected to handle. Perhaps an alternate way forward is to purposefully seek a more lightweight, high-utility, strategy that avoids trying to measure ‘everything’ and instead to focus on a small set of collectively agreed indicators that can be readily measured and reported. In addition, as Ling et al (2025) suggest, this approach would also seek to shift from traditional evaluation methods that seek causation, to more action-oriented approaches of discovery and learning [17]. Embedded research activity that uses theories of change to plan and deliver evaluations remain essential, but a learning health system model could be advanced where formal evaluation evidence is integrated alongside lived experiences, communities or practice, experiential learning and informed dialogue. Such an approach is potentially more likely to propagate the knowledge and understanding of the impact of integrated care that many so desperately seek.
